# Social structural factors that shape assisted injecting practices among injection drug users in Vancouver, Canada: a qualitative study

**DOI:** 10.1186/1477-7517-7-20

**Published:** 2010-08-31

**Authors:** Nadia Fairbairn, Will Small, Natasha Van Borek, Evan Wood, Thomas Kerr

**Affiliations:** 1 British Columbia Centre for Excellence in HIV/AIDS, St. Paul's Hospital, University of British Columbia, 608-1081 Burrard Street, Vancouver, B.C., V6Z 1Y6, Canada; 2 Department of Medicine, University of British Columbia, 10203-2775 Laurel Street, Vancouver, B.C., V5Z 1M3, Canada

## Abstract

**Background:**

Injection drug users (IDU) commonly seek manual assistance with illicit drug injections, a practice known to be associated with various health-related harms. We investigated the social structural factors that shape risks related to assisted injection and the harms that may result.

**Methods:**

Twenty semi-structured qualitative interviews were conducted with IDU enrolled in the ACCESS or Vancouver Injection Drug Users Study (VIDUS) who reported requiring assistance injecting in the past six months. Audio-recorded interviews were transcribed verbatim and a thematic analysis was conducted.

**Results:**

Barriers to self-injecting included a lack of knowledge of proper injecting technique, a loss of accessible veins, and drug withdrawal. The exchange of money or drugs for assistance with injecting was common. Harms experienced by IDU requiring assistance injecting included theft of the drug, missed injections, overdose, and risk of blood-borne disease transmission. Increased vulnerability to HIV/HCV infection within the context of intimate relationships was represented in participant narratives. IDU identified a lack of services available for those who require assistance injecting, with notable mention of restricted use of Vancouver's supervised injection facility.

**Conclusions:**

This study documents numerous severe harms that arise from assisted injecting. Social structural factors that shape the risks related to assisted injection in the Vancouver context included intimate partner relations and social conventions requiring an exchange of goods for provision of injecting assistance. Health services for IDU who need help injecting should include targeted interventions, and supervised injection facilities should attempt to accommodate individuals who require assistance with injecting.

## Introduction

The injection of illicit drugs is a growing public health concern internationally, and human immunodeficiency virus (HIV) transmission among injection drug users (IDU) represents a significant factor driving the global HIV epidemic. There are an estimated 16 million individuals who inject illicit drugs worldwide and 3 million injectors living with HIV [1]. Even in settings where a comprehensive public health response to injection drug use has been implemented, including needle exchange and health outreach programs, IDU continue to be exposed to a range of drug-related harms [2,3].

Recent studies have demonstrated that, even when sterile needles are accessible, individual characteristics and social structural factors may make IDU vulnerable to syringe sharing and subsequent HIV infection [2,4]. Rhodes' risk environment framework has identified a host of factors beyond the individual level that shape drug injecting practices and has illustrated how social context influences the production of injection-related HIV risks [5]. Social structural factors that may compromise individual ability to employ HIV prevention strategies among IDU include the influence of extended peer networks [6], as well as prevailing social norms among local populations of IDU [7]. Situated cultural norms have been shown to be particularly significant in shaping local and context-specific drug use risk practices, including routes of administration and"rituals" of use including drug procurement, exchange, and sharing [8]. Ethnographic research has highlighted the importance of the"cultural logics" of the street economy [9,10], as well as the gendered dynamics that often surround the injection process [11,12], as contextual factors that compromise individual ability to enact risk reduction strategies.

Previous work has indicated that a substantial proportion of IDU in various settings internationally receive manual assistance with injections [13,14]. The role of 'hit doctor' (i.e., someone who provides assistance with injections) was first described by Murphy (1991) who observed that experienced injectors working in shooting galleries in the San Francisco Bay area often provided assistance with injecting in exchange for money [15]. In Vancouver, Canada, a city with high rates of injection drug use and HIV among IDU, nearly half of local IDU have reported receiving assistance with injecting in the previous six month period [16]. In this setting, receiving assistance with injecting has been identified as a strong independent predictor of syringe sharing and HIV seroconversion, with IDU who report this behaviour being twice as likely to acquire HIV in comparison to IDU who do not require assistance injecting [16,17]. Assisted injection has also been associated with non-fatal overdose among IDU in Vancouver [18].

Given that assisted injection is a highly prevalent practice known to be associated with severe health complications in our setting, including HIV infection and overdose, we conducted a qualitative study to explore the circumstances and social conventions surrounding assisted injection. We sought to pay particular attention to individual factors as well as the broader contextual forces that shape the experience and harms of assisted injection.

## Methods

This article presents analyses of data from qualitative interviews with Vancouver IDU who require assistance injecting. One-to-one in-depth interviews were conducted to explore the following topics: 1) injection-related knowledge and practices; 2) experiences of assisted injection; 3) the broader context of assisted injection, and 4) harmful experiences resulting from assisted injection.

We draw upon data from 20 in-depth qualitative interviews conducted during June and July, 2007. Interviewees were recruited from two cohort studies in Vancouver: the Vancouver Injection Drug Users Study (VIDUS), which is composed of over 1000 HIV negative IDU; and ACCESS, which is composed of over 500 HIV-positive IDU. Database markers were used to identify participants from these cohorts who reported receiving assistance with injecting. Given the large representation of female IDU requiring assistance injecting in our setting [13,17], attempts were made to recruit female IDU for the present study.

Interviews were undertaken by three different trained interviewers (Fairbairn, Van Borek, and Small) and facilitated through the use of a topic guide encouraging discussion of assisted injection. Interviews lasted between 30 and 60 minutes, were tape-recorded, and were later transcribed verbatim. The research team discussed the content of the interview data throughout the data collection process, thus informing the focus and direction of subsequent interviews as well as developing a coding scheme for partitioning the data categorically. The content of transcribed interviews was catalogued using a coding framework specific to assisted injection and our analysis explores themes that emerged throughout the interviews. Two members of the research team (Fairbairn and Borek) separately catalogued the transcribed interview data using a coding framework, thus allowing for discussion of areas of agreement and instances of divergence.

All participants in the qualitative study provided informed consent to participate, and the study was undertaken with appropriate ethical approval granted by the Providence Health Care/University of British Columbia Research Ethics Board. There were no refusals of the offer to participate in the interview and no dropouts during the interview process. All interviewees received CDN$20 for their participation.

## Results

The study sample consisted of 20 participants, (7 male and 13 female) who ranged in age from 24 years to 51 years (median age = 40). Participant accounts described the potential barriers to self-injection, namely lack of knowledge of injection techniques or difficulty accessing veins due to long-term injecting. Social and structural factors that shape risk among IDU who require assistance with injecting were described by participants, including intimate partner relationships as well as the drug scene role of 'hit doctors' that require an exchange of goods for the provision of assistance injecting. Numerous harmful experiences that can result from assisted injection, namely increased risk for overdose and infectious disease transmission, were represented in participant narratives. One significant barrier to accessing care and support described by participants who require assistance with injecting was the rule prohibiting assisted injection at Vancouver's supervised injection facility (SIF).

### 1. Injection-Related Knowledge and Practices

#### a. Reasons for Requiring Assistance with Injecting

The accounts of interview participants indicate that several barriers prohibit individuals from being able to self-inject. Several participants described requiring assistance with injections because they lacked the injection-related knowledge necessary to self-inject, particularly at the time of their first injection.

I was thirteen years old and I was running away from a group home. And my best friend, we were watching her cousin,... and taking license plates, for when she was working on the street. So at the end of the night, we go back to her hotel room and she would shoot us some coke, for taking license plates and that... So she would fix herself and then she would fix me. (Female Participant #19)

Individuals described a loss of accessible veins, due to long-term injecting, as another key barrier to self-injection.

Well, it's veins, I have no veins. It's all collapsed, or calloused.... So there are times I can, every once in awhile a vein will pop up, and then I can use it for a few times, and then it will go back down.... If I can't get it in two or three times, there's somebody I know, you know he can get me in the arm or in my neck. (Male Participant #6)

Several participants described requiring assistance injecting due to collapsed veins and choosing to"jug" (inject in the jugular vein) in these instances.

I: You do mostly your own injections?

R: Ah, actually my boyfriend does it now, because sometimes I'm having a hard time with my arms now, because of all the injecting I did. Having to find a vein, he jugs me now {.....} Yeah, I can't find, like you know just can't find any veins sometimes, so he'll go in the neck for me, or I go myself in the neck. (Female Participant #2)

Some participants required assistance with injecting on occasion while experiencing symptoms of shakiness or feelings of anxiety, such as during instances of drug withdrawal.

Well I'm just being, I'm just being really anxious lately. I don't always need help, but I just want to have that hit. I want to have it... I don't want to fuck around anymore, my veins are pissing me off. (Female Participant #9)

One participant described his inability to self-inject due to a physical disability that prohibited him from using one of his arms.

My brother, my best friend, usually ties me off and does it because I have a disability... I can't because of the handicap. (Male Participant #14)

Within the context of intimate partner relationships, several female participants described assisted injection as a way to demonstrate trust and intimacy in addition to a form of needed assistance owing to a lack of injection-related knowledge or technique.

Yeah my partners have all you know... they fixed right. And usually they... it's a trust thing again. Kind of the more you know a person, then they know your body, how your veins are and stuff right. So it just works better that way it seems right, you know. Yeah, yeah and having that bond is also special too, which is cool. You know like you care, right, you don't want to hurt them, you don't want them to get hurt you know?{.....} Yeah, but usually, it's, my boyfriend will do both of us or whatever, yeah.{....}If he's a little sick, he might do himself first or whatever. But usually, I go first. (Female Participant #16)

I: The only person you ever had jug you was your husband?

*R: Yeah, and then I'd do it for him*.

I: He also had trouble with his veins?

R: Ah no, it was just that it was easier for me to do it for him, because I was already high, and he wanted to be high at the same time as me, so he'd fill it out, and I'd get high, and do him right away. (Female Participant #10);

#### b. Exchange for Assisted Injection Services

Participants described the provision of assisted injection services as a well-established role within the street economy that typically involves an exchange of money or drugs.

*R: If they're going to fix me with a ten paper of powder, I'd shoot them five bucks*.

I: Okay and always you give something?

R: Always... It's kind of like a cardinal rule down here. (Female Participant #19)

The amount of money or drugs exchanged for help with an injection varied and was negotiated between individuals. One participant described a willingness to pay more money when feeling a greater sense of urgency to use drugs.

He likes his rock. I'll give him the money to go buy it, or I'll just give it to him. I mean, he doesn't ask for it... If I want to get high real bad, it's worth a lot {....} once I get it in me, and I get the rush, it's worth a million dollars. (Male Participant #6)

Participants described the harms that can arise when 'hit doctors' have material incentive to help someone inject and may lack concern for preserving the safety of the individual they are injecting.

*It's *[assisted injection] *pretty risky, because you really don't know, they could be bullshitting you right? Because just to make that extra dollar or whatever.... (Female Participant #4)*

Because of this risk, participants emphasized the importance of having a trusting interpersonal relationship with a 'hit doctor'.

I've had people that like, ok, like, last night, I said"M I need your help." He goes"What's in it for me?" I said" Absolutely nothing" until today and then I get him back. But a lot of times, they see if they're being paid for it {....} But anyways last night... I was saying,"I need you, and I know you can do this, but how are you feeling"? He's got bad eyesight, and he can't buy glasses but I trust him, the trust factor is first. And then it's the physical, could he do it and see it? (Female Participant #9)

### 2. Harmful Experiences Due to Assisted Injection

Participants identified several potential harmful outcomes that can arise from relinquishing control over the injection process. These included missed injections and consequent health problems, robbery, infectious disease transmission, and overdose.

#### a. Missing the Injection

A variety of health complications including abscess formation and other forms of infection can result from missed injections. The most harmful complications of missed injections described by participants involved jugular injection, where the carotid artery, jugular vein, trachea, and recurrent laryngeal nerve are in close proximity to the point of the syringe [19].

Yeah, missing my shot in the neck. That was the scariest part, it was like a sharp pain right up to my head, and I was numb on this side for the longest time. {....} He just missed me, and I don't know, must have hit a, I don't know, he hit something.... I got scared, like I thought I was going to be gone or something, you know. (Female Participant #4)

The worst experience I had was before I got the abscess at the back of my throat, when somebody was jugging me, and somebody kicked the fucking, kicked me while I was getting jugged.{....} And then so two days later, an abscess formed in the back of my throat, right here.{....} and I almost died... because it formed so fast, and so quickly. {...} and it was starting to block my swallowing, and my breathing. (Female Participant #9)

#### b. HIV/HCV Transmission

Having one's syringe unknowingly exchanged (by the person providing assistance with injecting) for another containing only water was reported by numerous participants. In addition to theft of the drug, concern was expressed about receiving an injection with a syringe of unknown origin.

Actually it was my boyfriend too. When he was a heroin addict he was really bad. [...] Yeah he, I asked him to fix me, like I had heroin for sale, and he was holding my dope for me, and I asked him to fix me up one, {....} and there I could see him shaking something, he was putting water in it {....} I busted him right, he was going to switch me. Yeah. And he got really mad and threw my rig, and threw my dope across the street because I busted him. He was going to gypsy switch [swap rigs for one filled with water] me. (Female Participant #4)

Many IDU described relying on a 'hit doctor' to provide injection equipment in addition to administering the injection, resulting in vulnerability to HIV and other infectious diseases.

I: Is there anything else that you worry about when you're going to have someone else fix you?

R: Well not just about them switching rigs, but you don't know if the rig that they're giving you has HIV in it or not. {....} Well, yeah, like two weeks ago I fixed with a rig that had blood in it just because I was that dopesick. (Female Participant #19)

Syringe sharing between intimate partners who provide assistance injecting one another was a potential route of infectious disease transmission described by several participants.

*R: When I first started fixing heroin. Yeah my boyfriend would jug me and that...., we had this big canister. We'd just throw our used rigs in there and usually when we'd wake up, if we were dopesick, we would just grab any rig out of the container*.

I: Okay and so, would you usually get injected first?

R: No, he would do himself first and then me. (Female Participant #19)

When I had a boyfriend he used to inject me, but he used to do bad things though, change the needles and stuff... especially in my neck, he'd just push it in, in my neck went like this, you know, but he'd switched the rig... I got Hepatitis C from him, 'cause he gave me his bloody fix one time, that's how I got Hep C. (Female Participant #5)

#### c. Overdose

Accidental overdose was commonly reported. Several participants described incidents that involved miscommunication over the amount of drug to be injected which lead to overdose.

I used to throw the whole half a gram in the spoon right, but I mixed up rigs, different rigs for each amount, like 20 units in each one. I threw a half in there, and I turned my back, this other guy threw a ¼ gram in there and I didn't know about it... Boom, he fixed me, I got half way and I told him to stop, stop. I said,"No, no, don't do that". He was going on and on and he said,"It's okay, I'm almost there". I said,"No, no wait a minute". And boom, he pushed it in. I started vibrating, I was feeling like, Holy Shit. And I'm going"Oooh". Like I'm really starting to spin and everything. He's going,"are you okay". I said,"I'm okay, just don't touch me". He said,"No, no you need to get up and walk". And he grabbed me by the arm and pulled me up. I took two steps, and everything went white. (Male Participant #3)

This one girl she didn't tell me not to push it all in. .... So I smashed it all in and right after, before I pulled the needle out, she goes"You weren't supposed to put it all in" and then just, she just turned into like a robot. She was like, she started running, blindly, running into telephone poles, running into walls, into everything and just, holy smokes, I couldn't believe what was going on. (Female Participant #19)

### 3. Barriers to Injecting at the SIF

A number of participants described the rule prohibiting assisted injection at Vancouver's SIF as a barrier to engaging in safe injecting practices. The SIF is a place where IDU can inject pre-obtained illegal drugs under the supervision of nurses trained to provide an emergency response in the event of overdose. Presently, only verbal direction and limited manual assistance (excluding the act of injecting) is permissible from staff. Some participants noted that this assistance enabled them to administer their own injection, while others were still unable to self-administer their injection. Many of these individuals reported that they had to then leave the facility to find another IDU in the nearby alleys to assist with the injection.

I: Have there ever been times when you've needed to get some help with an injection, and you couldn't find somebody to help you out?

*R: Yeah. I had to really take my time to, I had to get Insite to help me, to direct me, because I couldn't find nobody else that was safe. {....} Yeah, I had to do it myself and eventually, I got it. {....} They just directed me, like you know, like telling me... which way to go. {...} Yeah, they talked me through it*.

I: Have you ever gone in there to fix, and then not been able to get it done yourself?

R: Yeah, I have. Go outside and see somebody there to jug me, yeah, that has happened. (Male Participant #2)

R: Like actually last night, it was so weird, I go, 'Well, I'm going to go inject, and then come back in here [InSite]'. It's because I hadn't been able to inject myself properly, and so I needed somebody to jug me and you can't get any assistance at all and sometimes I just can't take the time out with myself, I can't be with myself enough to actually inject myself properly and fast. Like 'cause I want to get it in me too fast, I get too anxious, 'get in'. And now, that's why I end up with shit like this [injection-related infection] on my arm, right? (Female Participant #4)

Some individuals reported that they would not use the SIF because of the rule prohibiting assisted injection.

I: What about that rule at INSITE where you can't get help with an injection?

R: That's the reason why I won't go there. I think that sucks. That, it's not good, it's, they should do something about something like that. 'Cause what happens if I want to go in there, and need help and nobody will help me? Well what's this place here for then? (Male Participant #5)

## Discussion

We identified a range of individual, social, and structural factors that shape the context of risk associated with assisted injection. The perspectives of participants in the present study highlight several barriers to self-injection, including lack of injection-related knowledge and technique, inability to access veins due to long-term injecting and physical disability. We documented a variety of harms that can result from relinquishing control over the injection process and identified various social factors that shape these harms, including intimate partner relations and social conventions requiring an exchange of goods for provision of injecting assistance. The rule prohibiting assisted injection at Vancouver's SIF was identified as a structural barrier to receiving injection-related instruction and support.

Participant accounts detailing assisted injections highlight the difficulty in ensuring that a syringe is sterile when obtaining assistance with injecting, and may help shed light on previous work that has found the characteristic of requiring assistance with injecting to be an independent predictor of HIV seroconversion [17]. Several participants in the present analysis described borrowing syringes and injection equipment from the 'hit doctor' when receiving assistance with injecting. These descriptions may help explain findings from previous research indicating that requiring help injecting is independently associated with reporting *borrowing *a used syringe and providing assistance injecting (e.g., being a 'hit doctor') is independently associated with *lending *one's own syringe [13,19]. Switching of syringes by the 'hit doctor' in order to steal drugs was commonly reported, and represents one important route by which HIV transmission may occur for individuals who receive assistance with injecting. Additionally, the finding that miscommunication and confusion surrounding the quantity of drugs administered may occur during assisted injections helps shed light on the previous epidemiological findings indicating that this practice is also associated with non-fatal overdose.

Narratives from several female participants portrayed assisted injection as an opportunity to share in the injecting process and drug high, thereby fostering an increased sense of trust and intimacy. Assisted injection as a symbolic act in the context of intimate relationships may therefore represent an important point of intersection of sexual and injecting dynamics, comprising a"dual risk" for HIV acquisition [20]. Previous qualitative work has investigated the gendered dynamics surrounding assisted injection by documenting women's experiences of theft and violence, including experiences of abuse from intimate partners when being injected with illicit drugs [12,21]. Though no such accounts were documented in our study, the gendered dynamics of assisted injection begs further exploration given that women are twice as likely as men to report requiring assistance with injecting in our setting [13,17].

IDU who require assistance with injecting unanimously reported an exchange of money or drugs in return for the provision of injecting assistance. This exchange of resources situates assisted injection services within the street economy and introduces the possibility of harm in instances where 'hit doctors' provide assistance with injecting purely for lucrative benefit [14,15]. This exchange-for-service dynamic may further exacerbate harms for IDU who require assistance with injecting by increasing the likelihood of violence resulting from disputes over compensation given the lack of an authority to resolve such disputes.

Vancouver's drug policy response to the ongoing HIV epidemic has involved the implementation of numerous harm reduction strategies including needle exchange programs, a heroin maintenance trial, and a SIF [22]. A current limitation of many SIFs, including the one in Vancouver, is that operational guidelines prohibit assisted injections on the premises due to concerns over civil liability should assisted injections be permitted within SIFs [23,24]. However, given the significant barriers to accessing care and the increased risk of HIV infection for individuals who require assistance with injecting, we recommend reconsideration of this policy. Indeed, a previous study of an unsanctioned drug-user-run SIF documented the successful implementation of an assisted injection policy, which resulted in many individuals developing the competency to self-inject [25].

The present study has several limitations that warrant acknowledgement. Firstly, our findings are based upon interviews with local IDU participating in the current study. While an effort was made to ensure that the study sample reflects the demographics of the local drug-using population who require assistance injecting, some perspectives may nonetheless be underrepresented. Secondly, as injection drug use is a highly stigmatized behaviour, it is possible that social desirability bias affected the responses of some participants. Thirdly, the data collected and analyzed here presents only the viewpoints of IDU; the results of this analysis should be compared with the findings of ethnographic research utilizing participant-observation within the SIF.

In summary, we found that barriers to self-injecting included a lack of knowledge of injection practices, symptoms of anxiety or withdrawal, or a loss of accessible veins. Our qualitative data indicate that numerous harms can result from the practice of assisted injection, notably increased risk for infectious disease transmission and overdose. Some women reported a preference to have a partner inject in order to develop trust and intimacy, underscoring the importance of considering social and contextual factors when examining infectious disease transmission among IDU. Participants identified the rule against assisted injection at the SIF to be a significant barrier to accessing health care, and therefore this policy should be re-evaluated.

## Competing interests

The authors declare that they have no competing interests.

## Authors' contributions

NF and TK were responsible for the study design and prepared the first draft of the analysis. NVB, WS, and EW assisted with the main content and provided critical comments on the final draft. All of the authors approved the final version submitted for publication.
